# Choroidal Circulation in Indolent, Nonprogressive, Multifocal Choroidal Lesions Evaluated by Multimodal Imaging Including Laser Speckle Flowgraphy

**DOI:** 10.3390/diagnostics16193163

**Published:** 2026-09-29

**Authors:** Yui Yamashita, Mizuho Mitamura, Michiyuki Saito, Kiriko Hirooka-Nishiyama, Susumu Ishida

**Affiliations:** Department of Ophthalmology, Faculty of Medicine and Graduate School of Medicine, Hokkaido University, N-15, W-7, Kita-ku, Sapporo 060-8638, Japan; yamayui.21@huhp.hokudai.ac.jp (Y.Y.); m1i1z3u2ho25@gmail.com (M.M.); hirooka0516@huhp.hokudai.ac.jp (K.H.-N.); ishidasu@med.hokudai.ac.jp (S.I.)

**Keywords:** choroidal circulation in indolent, nonprogressive, multifocal choroidal lesions, laser speckle flowgraphy, indocyanine green angiography

## Abstract

**Background:** Indolent, nonprogressive, multifocal choroidal lesions (INMCLs) are low-grade B-cell lymphoid infiltration with a good visual prognosis, yet their impact on choroidal circulation remains poorly understood. **Clinical Significance:** We report two cases of INMCLs evaluated via multimodal imaging, focusing on circulatory dynamics using laser speckle flowgraphy (LSFG). **Case Presentation:** Two patients (a 71-year-old female and a 54-year-old male) with histories of glaucoma presented with multiple yellow-white choroidal lesions at the posterior pole. Optical coherence tomography revealed extensive hyporeflective choroidal spaces in the outer choroid, while indocyanine green angiography (ICGA) showed corresponding hypofluorescent spots. Significantly, LSFG demonstrated regional reduction in LSFG signal, visualized as a “cold colour” pattern extending from the optic disc to the macula. Although both patients exhibited glaucomatous visual field changes, the extent to which INMCL contributed to the paracentral defects remains uncertain and difficult to separate from advanced glaucomatous damage. The clinical findings remained stable over several years without treatment. **Conclusions:** Our multimodal analysis leads to the tentative hypothesis that INMCLs may be associated with choroidal circulation disturbances, which could putatively stem from mechanical vascular compression due to localized lymphocyte accumulation. These cases highlight the potential utility of longitudinal LSFG in monitoring regional choroidal hemodynamics in INMCL, although its capability to differentiate INMCLs from other choroidal infiltrative diseases warrants validation in future comparative cohorts.

## 1. Introduction

Indolent, nonprogressive, multifocal choroidal lesions (INMCLs) are characterized by low-grade B-cell lymphoid infiltration first described as unilateral, yellow-white choroidal lesions typically distributed around the macula and peripapillary area [1,2,3]. It has hitherto been known that patients with primary choroidal lymphoma rarely go on to develop systemic lymphoma [4], and it was proposed to distinguish between primary and secondary choroidal lymphoma because of their different clinical and histological features, management, treatment and prognosis. Of patients diagnosed with INMCLs [2], histopathology proved to be composed of low-grade small B-cell lymphoma cells after 19 years of long-term follow-up [3]. It is recommended that biopsy should not be performed so that they remain confined to the choroid for long periods, with a good visual prognosis [2,3]. Regarding multimodal imaging of INMCL, optical coherence tomography (OCT) typically reveals well-defined, uniform, hyporeflective spaces or vacuolation corresponding to the choroidal lesions, sparing the inner choroid [1,2,3,5,6]. Hypofluorescence on indocyanine green angiography (ICGA) [2,5,6] partially suggests a circulatory disturbance in the choroid; however, the details of choroidal circulation in INMCL are unknown.

Laser speckle flowgraphy (LSFG) is a laser scattering-based blood flow imaging device and a powerful tool that non-invasively delineates the fundus circulation in retino-choroidal disease in two dimensions. We have investigated the retino-choroidal circulation of various intraocular diseases, including central serous chorioretinopathy (CSC) [7,8], Vogt–Koyanagi–Harada (VKH) disease [9], sclerochoroidal calcification [10], choroidal lymphoma [11], and leukemic retinopathy [12,13]. Herein, we present two cases of INMCLs observed with multimodal imaging, including evaluation of the choroidal circulation with LSFG.

## 2. Methods/Imaging Protocol

LSFG (LSFG-NAVI, version 3.1.39.2, Softcare Ltd., Fukuoka, Japan) images were acquired under standardized conditions: pupil dilation using topical 0.4% tropicamide (Mydrin-M; Santen Pharmaceutical Co., Ltd., Osaka, Japan), stable systemic blood pressure and pulse rate, controlled intraocular pressure (IOP), and fixed acquisition times. Ophthalmic examinations were conducted after pupils were fully dilated and devoid of light reflex. For each examination, 4 to 5 consecutive raw scans meeting system quality and fixation criteria were obtained.

For wide-field qualitative visualization, multiple high-quality images obtained on the same day were montaged manually across the posterior pole. Because stitching boundaries introduce focal variations in illumination and scattering intensity, montaged composites were strictly restricted to qualitative assessment (Figure 1h and Figure 2h).

For quantitative hemodynamic evaluation, non-montaged, single-field raw images were analyzed using an LSFG Analyzer (version 3.0.47.0) across longitudinal follow-up visits (9 visits over 28 months in Case 1; 10 visits over 23 months in Case 2). Three standardized regions of interest (ROIs) were defined on the fundus map (Figure 3a): (1) the fovea (blue circle), encompassing macular microvasculature and underlying choroidal vessels; (2) the PPA area involved by the INMCL lesion (red circle); and (3) an adjacent normal choroidal area temporal to the macula devoid of both PPA and INMCLs (purple circle). Notably, unlike the foveal ROI, both the PPA and normal area ROIs were meticulously positioned to avoid major retinal and choroidal vessels, thereby isolating choroidal tissue mean blur rate (MBR) and enabling reliable comparability between eyes. Longitudinal ROI positioning across visits was co-registered using the software’s automated alignment tool and manually fine-tuned by referencing stable retinal vascular bifurcation landmarks. The coefficient of variation (CV) across consecutive scans within each session was calculated to evaluate measurement repeatability. To rule out measurement error without pseudoreplication, mean visit values were utilized to compare lesional versus non-lesional and fellow-eye parameters.

## 3. Case Presentation

### 3.1. Case 1

A 71-year-old woman was referred to our hospital for detailed examination of a small yellow-white choroidal lesion in the oculus sinister (OS), which had been noted during a regular check-up for glaucoma. She was diagnosed with normal-tension glaucoma at age 63 and was treated with glaucoma eye drops (ripasudil hydrochloride, bimatoprost). Her IOP was controlled between 8 and 11 mmHg. She was being followed up for a brain aneurysm. There was no significant family history. At the initial examination, her best-corrected visual acuity (BCVA) was 0.7 oculus dexter (OD) and 0.2 OS with myopic astigmatism and intraocular lens oculus uterque (OU). Slit-lamp microscopy did not detect any anterior chamber inflammation OU. Colour fundus photography (CFP) showed small, partially fused yellow-white spots within the posterior pole and the upper optic disc OS (Figure 1a). Cup/Disc (C/D) ratio was 0.9 OD and full cupping OS. Fundus autofluorescence (FAF) detected no obvious abnormalities, confirming that the retinal pigment epithelium (RPE) and retina were preserved (Figure 1b). Swept-source OCT (SS-OCT) (DRI OCT Triton; Topcon Inc., Tokyo, Japan) demonstrated the extensive hyporeflective choroidal spaces within the outer choroid throughout almost the entire posterior pole (Figure 1c, arrows), corresponding to fundus lesions, together with hyperreflective areas within the choriocapillaris (Figure 1c, arrowheads). Retinal structures above Bruch’s membrane were preserved and no intraocular inflammation was observed. The mean ganglion cell complex (GCC) thickness OD and OS were 56.50 ± 7.78 μm and 51.50 ± 0.71 μm, respectively. Significant thinning of both the peripapillary RNFL and macular GCC was observed on SS-OCT, particularly in the superior sectors, which corresponded with the localized visual field defects. Fluorescein angiography (FA) revealed hyperfluorescent spots consistent with CFP from the early to late phases without fluorescence leakage (Figure 1d,e, dotted lines). ICGA showed hypofluorescence from the early to late phases (Figure 1f,g, dotted lines). LSFG exhibited regional reduction in LSFG-MBR signal represented by a cold colour pattern consistent with CFP (Figure 1h, choroidal lesions: yellow dotted lines), especially from the optic disc to the macula except the area of parapapillary chorio-retinal atrophy (PPA) (Figure 1h, PPA: white dotted lines). Humphrey Field Analyzer (HFA) 30-2 testing of the right and left eyes revealed mean deviation (MD) values of −18.08 dB and −16.55 dB, pattern standard deviation (PSD) values of 15.75 dB and 11.13 dB, and visual field index (VFI) values of 37% and 39%, respectively. Goldmann perimetry detected visual field defects from the optic disc to the superior Bjerrum and macular regions with grade III-a by Kozaki’s classification OS (Figure 1i) and grade III-a by Kozaki’s classification OD, respectively.

Quantitative LSFG analysis across 9 longitudinal visits (August 2020 to December 2022) revealed high measurement repeatability (intra-session mean CV < 8.5%). In the non-lesional normal choroidal area (purple circle), tissue MBR was comparable between the affected and fellow eyes (5.94 ± 1.16 vs. 6.22 ± 0.86, ratio: 0.95), confirming that baseline systemic and ocular perfusion were equivalent. In contrast, tissue MBR in the lesion-involved PPA area (red circle) in the affected eye showed a marked, 69.2% reduction compared with the fellow eye’s PPA (1.70 ± 0.52 vs. 5.52 ± 0.69, ratio: 0.31) and a 71.4% reduction compared with the adjacent normal choroid in the same eye (ratio: 0.29) (Figure 3). Foveal MBR (blue circle) was also reduced in the affected eye (4.53 ± 0.81 vs. 11.72 ± 1.63, ratio: 0.39). Over the 28-month follow-up, MBR values across all three ROIs remained stable without longitudinal decline (linear regression trend, *p* > 0.10).

After confirming the absence of systemic abnormalities through examinations including a whole-body computed tomography (CT) scan, the patient was diagnosed with INMCLs based on the multimodal imaging characteristics of multiple yellow-white choroidal lesions clustered in the posterior pole that corresponded to hypofluorescent and hyporeflective areas on ICGA and OCT, respectively, and was observed with no treatment. Her ophthalmological findings remained unchanged for 3 years 10 months after the diagnosis.

### 3.2. Case 2

A 54-year-old man was referred to our hospital for blurred vision that persisted before and after cataract surgery. He was diagnosed with open-angle glaucoma at age 42 and treated with glaucoma eye drops (latanoprost and timolol maleate ophthalmic solution, brimonidine, and dorzolamide). His IOP was controlled between 10 and 15 mmHg. His medical history included hypertension and polycythemia. He underwent a living kidney transplant for polycystic kidney disease three years ago and has continued immunosuppressive therapy with oral prednisolone 4 mg/day, mycophenolate mofetil 250 mg/day, and tacrolimus 1.5 mg/day; his kidney function has been maintained at a serum creatinine level of 1.37 mg/dL and an estimated glomerular filtration rate of 43.1 mL/min/1.73 m^2^. To rule out post-transplant lymphoproliferative disorder and systemic lymphoma, he underwent monthly blood tests, electrocardiograms every two months, and a full-body CT scan once a year. There were no special notes on family history. At the initial examination, his BCVA was 0.8 OD and 0.5 OS with myopic astigmatism and intraocular lens. Slit-lamp microscopy did not detect any anterior chamber inflammation OU. CFP showed multiple yellow-white spots extending from the optic disc to the macula OS (Figure 2a). C/D ratio was 0.9 OD and full cupping OS. FAF detected slight hypoautofluorescence below the macula OS (Figure 2b). SS-OCT demonstrated the extensive hyporeflective choroidal spaces within the outer choroid ranging between upper and lower vascular arcades (Figure 2c, arrows) together with hyperreflective area within the choriocapillaris OS (Figure 2c, arrowheads). The mean GCC thickness OD and OS were 55.5 ± 4.95 μm and 56.75 ± 4.35 μm, respectively. Significant thinning of both the peripapillary RNFL and macular GCC was observed on SS-OCT, particularly in the inferior sectors, which corresponded with the localized visual field defects. FA revealed hyperfluorescent spots with partly hypofluorescence surrounding the optic disc (Figure 2d,e, dotted lines), and ICGA showed hypofluorescence from the early to late phases OS (Figure 2f,g, dotted lines). LSFG images were combined from multiple acquisitions on the same day to include the lesion area, selecting the highest quality images. LSFG displayed regional reduction in LSFG-MBR signal, indicated by a cold colour pattern below the optic disc and extending from optic disc to macula consistent with CFP (Figure 2h, choroidal lesions: yellow dotted lines) except the PPA area (Figure 2h, PPA: white dotted lines). HFA 30-2 testing of the right and left eyes revealed MD values of −12.07 dB and −7.27 dB, PSD values of 12.06 dB and 9.60 dB, and VFI values of 65% and 75%, respectively. Goldmann perimetry revealed visual field defects from the optic disc to the inferior Bjerrum and macular regions with grade II-b by Kozaki’s classification OS (Figure 2i) and grade II-b by Kozaki’s classification OD, respectively.

Quantitative longitudinal LSFG evaluation across 10 visits (April 2021 to March 2023) demonstrated a virtually identical pattern (Figure 3). Non-lesional normal choroidal MBR was preserved (5.40 ± 0.55 OS vs. 6.48 ± 0.88 OD, ratio: 0.83). The lesion-involved PPA area (red circle) exhibited a profound 58.0% reduction compared with the fellow eye’s PPA (1.85 ± 0.43 vs. 4.40 ± 0.58, ratio: 0.42) and a 65.7% reduction compared with the ipsilateral normal choroid (ratio: 0.34). Foveal MBR (blue circle) was likewise suppressed (4.51 ± 0.69 vs. 7.29 ± 1.34, ratio: 0.62). The hemodynamic parameters remained completely stable over the 2-year surveillance period without progressive deterioration.

Given his transplant history, a comprehensive systemic and hematologic evaluation was performed to rule out post-transplant lymphoproliferative disorder and secondary choroidal lymphoma. This work-up included complete blood cell counts, serum biochemistry, whole-body CT scans, and close interdepartmental consultations with nephrology and hematology, all of which confirmed the absence of systemic malignancy, lymphadenopathy, or visceral involvement. After confirming the absence of systemic abnormalities, the patient was diagnosed with INMCL and was observed with no treatment. His ophthalmological findings remained unchanged for 3 years 8 months after the diagnosis.

The institutional review board of Hokkaido University waived ethical assessment of this clinical study because this study was a case report with a non-invasive study. This study adhered to the tenets of the Declaration of Helsinki.

## 4. Discussion

The present study demonstrated novel findings in INMCLs via multimodal imaging techniques, so as to better understand the pathophysiology of the disease. To the best of our knowledge, this report is the first to demonstrate LSFG-MBR signal reduction in INMCL using LSFG. Although a previous study addressed choroidal circulation in INMCL using OCT angiography (OCTA), the present findings add novel insights by evaluating two-dimensional regional hemodynamics via LSFG.

The diagnosis of INMCL in our cases was established based on established diagnostic criteria: the characteristic presentation of multiple yellow-white choroidal lesions clustered at the posterior pole, extensive hyporeflective choroidal spaces on SS-OCT, persistent hypofluorescence on ICGA, and long-term clinical indolence without treatment. Other differential diagnoses were carefully excluded. Sclerochoroidal calcification was ruled out by the absence of dense placoid calcific lesions. Secondary choroidal lymphoma was excluded through comprehensive systemic evaluations (whole-body CT and hematologic panels) and the lack of systemic progression or lymphoma manifestation over a multi-year follow-up. Birdshot chorioretinopathy was differentiated by the absence of anterior chamber or vitreous inflammation, lack of characteristic oval, and cream-coloured retinal/choroidal spots radiating from the optic nerve.

Generally, the underlying causative mechanisms of choroidal thickening are broadly divided into non-inflammatory and inflammatory etiologies. The former corresponds to the pathogenesis of CSC, and the latter to VKH disease. CSC, representative of a non-inflammatory pattern, presents choroidal vasodilation in Haller’s layer, i.e., the pachyvessel, which is shown as an enlarged hyporeflective luminal structure on OCT, and as a warm colour signal on LSFG [7,8]. In contrast, the inflammatory pattern typical of VKH is theorized to be associated with choroidal infiltration of leukocytes causing flow obstruction due to the adhesion of cells to the inner wall of vessels, followed by compression from outside the vessels surrounded by extravasated cells [9,11]. Choroidal inflammation causes hyperpermeability of the vessels, resulting in fluid accumulation in the choroidal stroma, and manifests as a dense hyporeflective choroidal thickening on OCT and reduced blood flow on LSFG [9].

However, there are diseases that cannot be classified into the above two patterns. The thickened choroid with impaired circulation on OCT and LSFG observed in leukemic retinopathy [12,13] and secondary choroidal lymphoma [11] has been named the pseudo-inflammatory pattern. Although the choroidal morphology and circulation resemble an inflammatory pattern, the former mechanism is due to the choroidal infiltration of abnormally proliferating leukemic cells, and the latter to the choroidal infiltration of neoplastic lymphoma cells, both of which have little involvement of inflammatory leukocytes. The present two cases of INMCLs would be another example of the pseudo-inflammatory pattern: the hyporeflective cavity with choroidal thickening on OCT was due to a localized choroidal accumulation of low-grade lymphocytes, and the cold colour signal on LSFG was not only due to the lack of the blood flow signal in the cavity per se, but also to the choroidal circulatory disturbance by vascular compression occurring outside of the cavity. These findings were supported by the previous histopathological observations [3] and were consistent with the partial blockage of fluorescence on ICGA [2,5,6] and reduced blood flow on OCTA [6] observed in previous reports. While previous multimodal imaging and OCTA reports have been limited to structural descriptions or capillary-level analysis, LSFG enables the non-invasive, repeated assessment of blood flow and is likely to contribute as an additional tool in the differential diagnosis of INMCL.

The quantitative findings in the present cases provide concrete hemodynamic evidence corroborating the “pseudo-inflammatory pattern” (Figure 3). By establishing standardized ROIs on single-field scans, we attempted to differentiate the vascular impact of PPA from that of INMCLs, although their complete separation remains challenging due to anatomical overlap. In the fellow eyes, PPA alone accounted for only a modest decrease in perfusion compared with normal choroid (11.3% reduction in Case 1; 32.1% in Case 2). However, the presence of INMCLs superimposed a substantial hypoperfusion, suppressing tissue MBR down to approximately 1.7–1.8 (reductions of 58–69% compared with fellow-eye PPA, and 66–71% compared with ipsilateral normal choroid). Because non-lesional normal choroidal perfusion was equivalent between eyes, this marked reduction cannot be attributed to systemic antihypertensive medications, glaucoma eye drops, or generalized perfusion shifts, but rather supports an association between lesion-affected areas and reduced LSFG-MBR signal.

Both cases in the present study were complicated by glaucoma and had limited BCVA, which differed from other cases of INMCLs reported to have good visual acuity [1,2]. Goldmann perimetry showed defects in the Bjerrum area and peripheral nasal step, characteristic of glaucoma, in both affected and fellow eyes. Although the paracentral visual field defects observed in the affected eyes appeared atypical for glaucomatous optic neuropathy alone, whether these atypical defects were directly influenced by the submacular choroidal lesions remains uncertain. Chujo et al. reported INMCLs with mild RPE impairment and found that photoreceptor function was preserved in that neither multifocal electroretinogram (mERG) amplitudes nor visual field thresholds were reduced [5]. The cause of the visual acuity loss and visual field defects in the present cases may be the combination of glaucomatous visual field defects and the influences of choroidal circulatory disturbance due to extensive hyporeflective choroidal spaces surrounding the optic nerve and located directly below the papillomacular bundle, which is larger than previously reported [1,2,5,6]. Although FAF showed minimal or subtle alterations corresponding to the lesions in Case 2, and given the previously reported generally good visual acuity and visual field, the contribution of INMCLs to functional loss remains uncertain.

There are some limitations in this report. First, this is a case report with a small sample size of two cases, necessitating further multi-centre cohorts. Second, neither patient underwent histopathological confirmation, and the concept of mechanical vascular compression remains a working hypothesis. Third, although we minimized measurement artifacts and demonstrated long-term stability by repeating standardized single-field acquisitions over 2 years, quantitative MBR reflects composite tissue backscattering; therefore, potential signal attenuation resulting from thickened lymphoid infiltrates or altered optical properties cannot be entirely separated from true hemodynamic compromise. Finally, because objective electrophysiological tests (such as multifocal ERG) were not performed, the precise contribution of outer retinal dysfunction to visual acuity and visual field loss remains to be validated. Although these findings remain hypothesis-generating, our observations provide valuable quantitative and qualitative evidence of localized, stable choroidal circulatory impairment in INMCL.

## 5. Conclusions

In conclusion, we have reported two cases of INMCLs with extensive choroidal lesions surrounding the optic nerve and located directly below the papillomacular bundle, which were complicated by pre-existing glaucoma. While our multimodal imaging analyses including LSFG provide complementary longitudinal information regarding regional hemodynamics in INMCLs, whether the observed choroidal circulation disturbance is directly associated with mechanical compression from lymphocyte accumulation remains a working hypothesis that requires validation in future comparative cohorts.

## Figures and Tables

**Figure 1:**
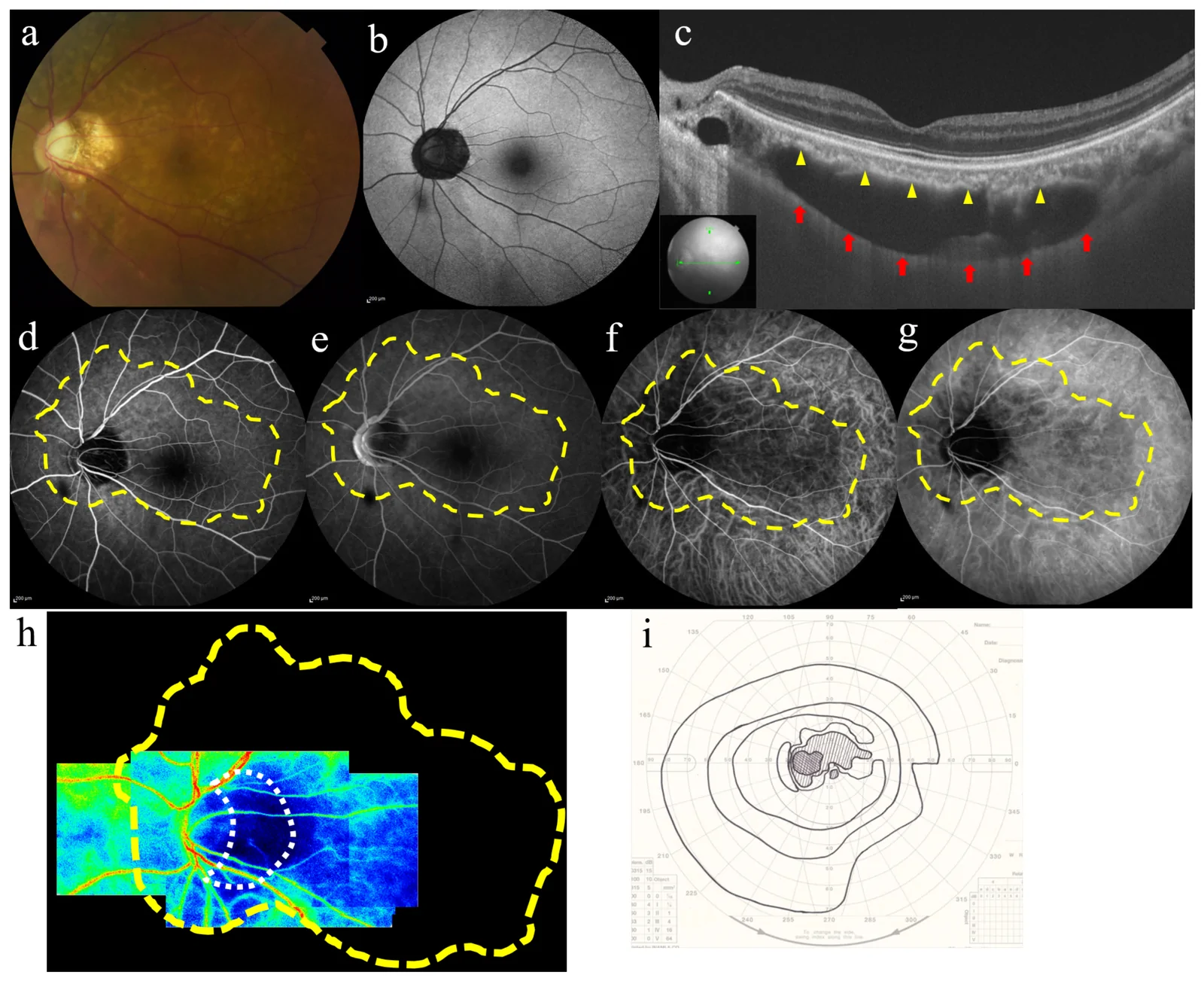
Clinical findings of the left eye in case 1 of indolent, nonprogressive, multifocal choroidal lesions. (**a**) Colour fundus photography shows small, partially fused yellow-white spots within the arcade and around the upper optic disc. (**b**) Fundus autofluorescence shows no obvious abnormalities. (**c**) Swept-source optical coherence tomography shows thickened hyporeflective choroidal spaces or vacuolation within the outer choroid (arrows) together with hyperreflective areas within the choriocapillaris (arrowheads). (**d**,**e**) Fluorescein angiography shows hyperfluorescent spots surrounding hypofluorescence from early (**d**) to late phases (**e**) (dotted lines). (**f**,**g**) Indocyanine green angiography shows hypofluorescence from the early (**f**) to late phases (**g**) (dotted lines). (**h**) Laser speckle flowgraphy shows a cold colour pattern from the optic disc to the macula. (**i**) Goldmann perimetry detected visual field defects from the optic disc to the superior Bjerrum and macular regions.

**Figure 2:**
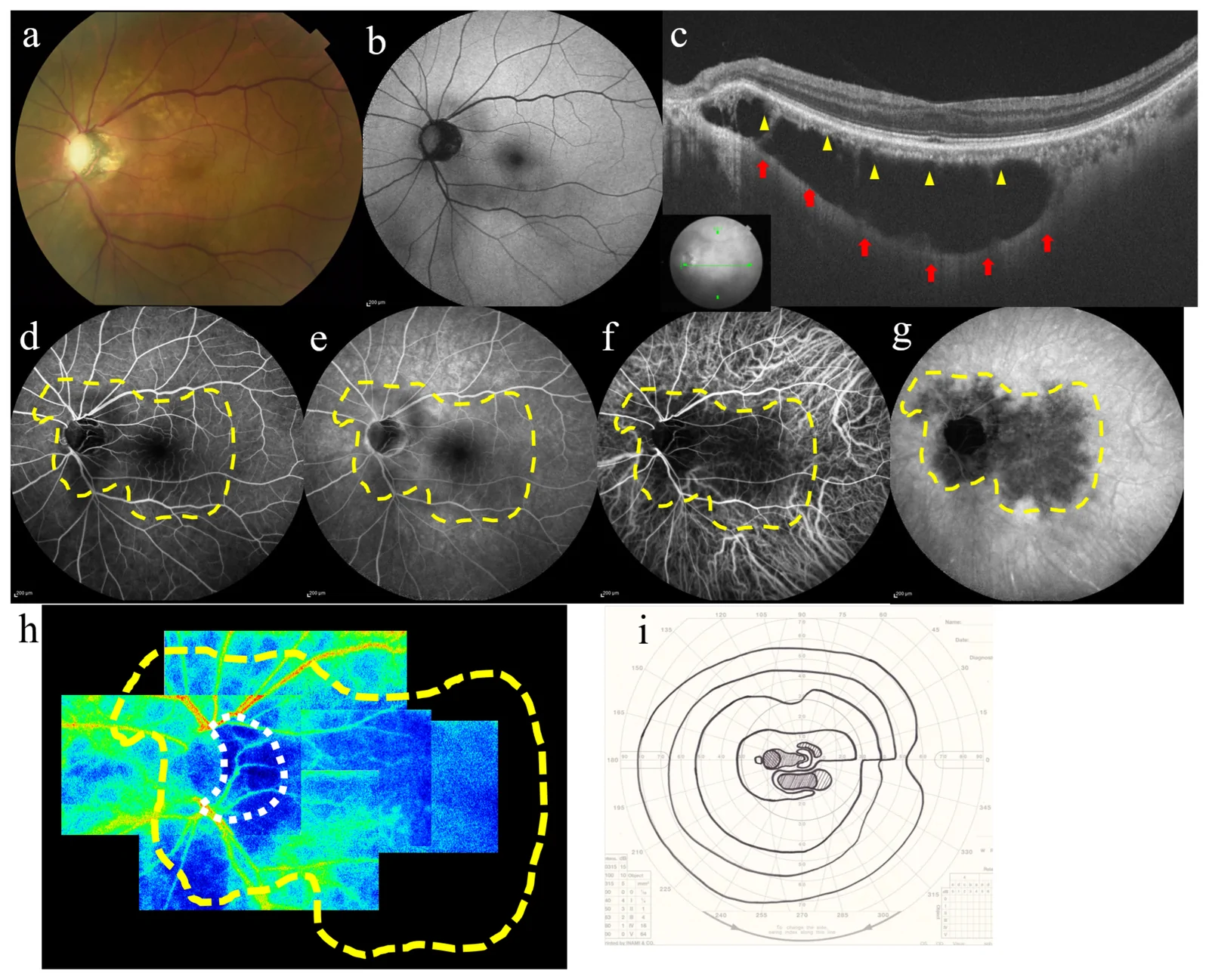
Clinical findings of the left eye in case 2 of indolent, nonprogressive, multifocal choroidal lesions. (**a**) Colour fundus photography shows small multiple yellow-white spots from the optic disc to the macula. (**b**) Fundus autofluorescence shows slight hypoautofluorescence below the macula. (**c**) Swept-source optical coherence tomography shows thickened hyporeflective choroidal vacuolation within the outer choroid (arrows) together with hyperreflective areas within the choriocapillaris (arrowsheads). (**d**,**e**) Fluorescein angiography shows hyperfluorescent spots from the early (**d**) to late phases (**e**) (dotted lines). (**f**,**g**) Indocyanine green angiography shows hypofluorescence from the early (**f**) to late phases (**g**) (dotted lines). (**h**) Laser speckle flowgraphy shows a cold colour pattern below the optic disc and extending from the optic disc to the macula. (**i**) Goldmann perimetry revealed visual field defects from the optic disc to the inferior Bjerrum and macular regions.

**Figure 3:**
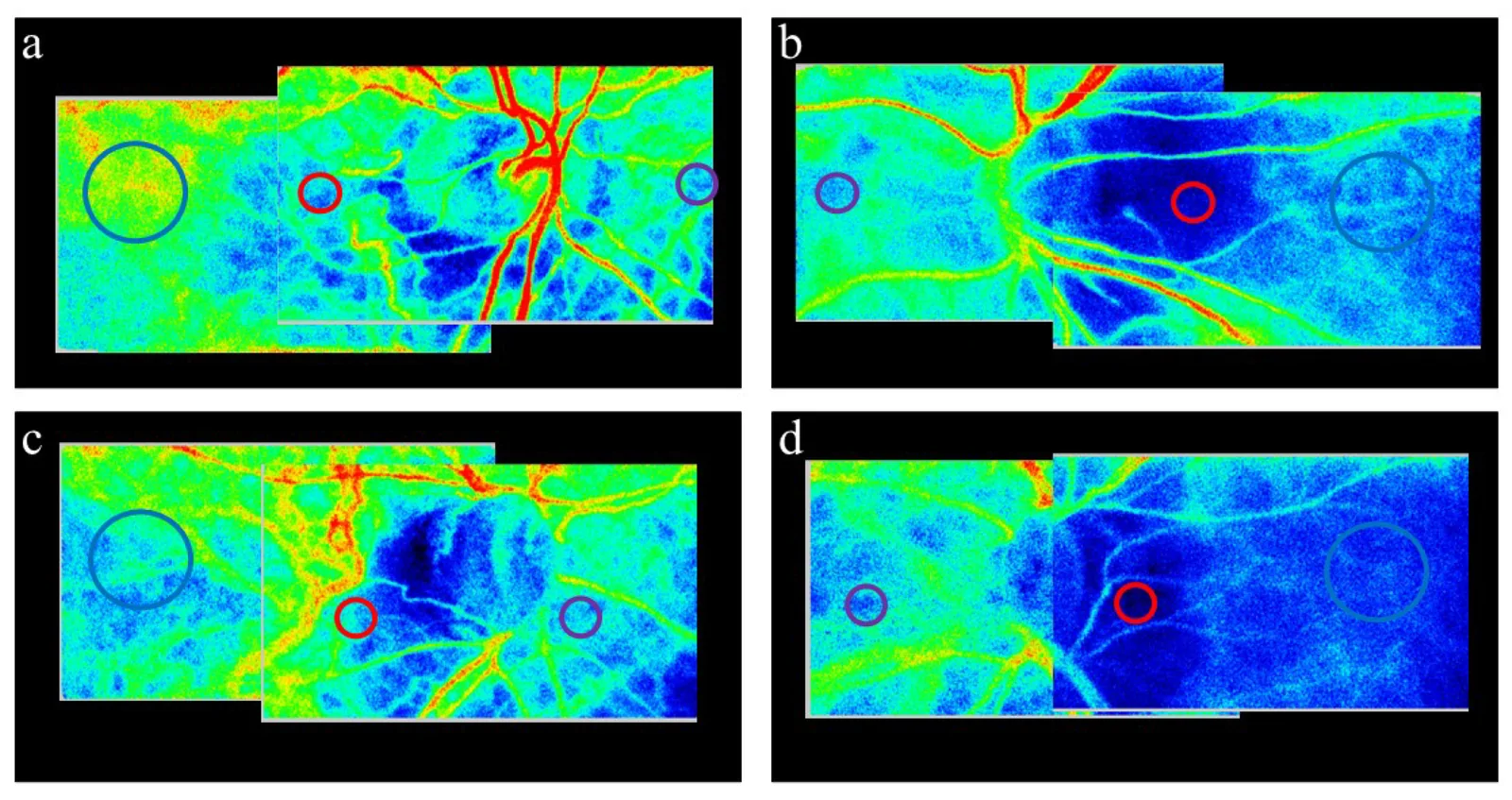
Quantitative evaluation of choroidal blood flow using single-field laser speckle flowgraphy (LSFG) maps in indolent, nonprogressive, multifocal choroidal lesions (INMCLs). Representative single-field LSFG colour maps of the fellow right eye in Case 1 (**a**), the affected left eye in Case 1 (**b**), the fellow right eye in Case 2 (**c**), and the affected left eye in Case 2 (**d**). Three standardized regions of interest (ROIs) are defined in each eye: the fovea (blue circle), encompassing macular microvasculature and large choroidal vessels; the peripapillary atrophy (PPA) region (red circle), positioned within the INMCL in the affected eyes (and the corresponding anatomical PPA in the fellow eyes) while avoiding major retinal vessels to measure choroidal tissue mean blur rate (MBR); and the non-lesional normal choroidal area temporal to the macula (purple circle), positioned in an area devoid of both PPA and INMCLs while avoiding major retinal vessels. In both patients, a selective and marked reduction in LSFG-MBR signal (cold colour pattern) is observed within the lesion-involved PPA area (red circle) in the affected eyes (**b**,**d**) compared with the adjacent normal choroid (purple circle) and the fellow eyes’ PPA (**a**,**c**). Conversely, non-lesional normal choroidal perfusion (purple circle) remains equivalent between the affected and fellow eyes.

## Data Availability

The data that support the findings of this study are available on request from the corresponding author, Y.Y. and M.S. The data are not publicly available due to their containing information that could compromise the privacy of research participants.

## Abbreviations

The following abbreviations are used in this manuscript:

INMCLs	Indolent, nonprogressive, multifocal choroidal lesions
LSFG	laser speckle flowgraphy
OS	oculus sinister
OCT	optical coherence tomography
OCTA	optical coherence tomography angiography
FA	fluorescein angiography
ICGA	indocyanine green angiography
CSC	central serous chorioretinopathy
VKH	Vogt-Koyanagi-Harada disease
BCVA	Best-corrected visual acuity
OD	Oculus dexter
IOP	intraocular pressure
OU	oculus uterque
CFP	colour fundus photography
FAF	fundus autofluorescence
RPE	retinal pigment epithelium
SS-OCT	swept-source OCT
mERG	multifocal electroretinogram
